# Interpretation of COVID-19 Epidemiological Trends in Mexico Through Wastewater Surveillance Using Simple Machine Learning Algorithms for Rapid Decision-Making

**DOI:** 10.3390/v17010109

**Published:** 2025-01-15

**Authors:** Arnoldo Armenta-Castro, Orlando de la Rosa, Alberto Aguayo-Acosta, Mariel Araceli Oyervides-Muñoz, Antonio Flores-Tlacuahuac, Roberto Parra-Saldívar, Juan Eduardo Sosa-Hernández

**Affiliations:** 1 School of Engineering and Sciences, Tecnologico de Monterrey, Monterrey 64849, Mexico; a01740937@tec.mx (A.A.-C.); orlando.delarosa@tec.mx (O.d.l.R.); alcubojalmex@gmail.com (A.A.-A.); maao@ssi.dk (M.A.O.-M.); antonio.flores.t@tec.mx (A.F.-T.); 2 Institute of Advanced Materials for Sustainable Manufacturing, Tecnologico de Monterrey, Monterrey 64849, Mexico; 3 Virology & Microbiological Preparedness, Statens Serum Institut, Artillerivej 5, 2300 Copenhagen, Denmark; 4 Biomolecular Innovation Group, Facultad de Agronomía, Universidad Autónoma de Nuevo León, Francisco Villa S/N, Col. Ex Hacienda El Canadá, General Escobedo 66415, Mexico; ibqrps@gmail.com; 5 Magan Centre of Applied Mycology (MCAM), Faculty of Engineering and Applied Sciences, Cranfield University Cranfield, Cranfield, Bedford MK43 0AL, UK

**Keywords:** SARS-CoV-2, wastewater surveillance, machine learning, data-based decision-making

## Abstract

Detection and quantification of disease-related biomarkers in wastewater samples, denominated Wastewater-based Surveillance (WBS), has proven a valuable strategy for studying the prevalence of infectious diseases within populations in a time- and resource-efficient manner, as wastewater samples are representative of all cases within the catchment area, whether they are clinically reported or not. However, analysis and interpretation of WBS datasets for decision-making during public health emergencies, such as the COVID-19 pandemic, remains an area of opportunity. In this article, a database obtained from wastewater sampling at wastewater treatment plants (WWTPs) and university campuses in Monterrey and Mexico City between 2021 and 2022 was used to train simple clustering- and regression-based risk assessment models to allow for informed prevention and control measures in high-affluence facilities, even if working with low-dimensionality datasets and a limited number of observations. When dividing weekly data points based on whether the seven-day average daily new COVID-19 cases were above a certain threshold, the resulting clustering model could differentiate between weeks with surges in clinical reports and periods between them with an 87.9% accuracy rate. Moreover, the clustering model provided satisfactory forecasts one week (80.4% accuracy) and two weeks (81.8%) into the future. However, the prediction of the weekly average of new daily cases was limited (R^2^ = 0.80, MAPE = 72.6%), likely because of insufficient dimensionality in the database. Overall, while simple, WBS-supported models can provide relevant insights for decision-makers during epidemiological outbreaks, regression algorithms for prediction using low-dimensionality datasets can still be improved.

## 1. Introduction

SARS-CoV-2, a novel coronavirus causing respiratory illnesses of varying severity in humans (denominated as COVID-19), was first detected in December 2019 and spread rapidly across the world, leading to the declaration of a worldwide emergency by the World Health Organization (WHO) by March 2020 [1]. Despite widespread adoption of preventive measures, including social distancing, reduction in in-person activities in schools and workplaces, cancelation of massive events, mandatory usage of facemasks, and extensive vaccination efforts, COVID-19 prevalence stayed stubbornly high throughout the world during 2020, 2021, and 2022, as the WHO would not declare the end of the sanitary emergency until May 2023 [2]. Incidence of COVID-19 cases showed a highly fluctuating behavior due to the continued emergence of new variants, driven by the high mutation rate of the viral RNA genome and the process of adaptation to both human immune responses and the deployment of vaccines and therapeutic agents [3]. While the definition of “wave” may vary depending on different criteria or geographical regions, between six [4] and seven [5] waves were registered between January 2020 and March 2023, with varying levels of intensity depending on the degree of infectivity of the underlying variants of concern. A total of 775,615,722 cases were reported worldwide by June 2024, although limited testing indicates that the actual incidence must be higher [6].

As a part of epidemiological containment and prevention efforts undertaken by public health authorities, there has been a growing interest in modeling the spread of the disease within populations [7]. The main approaches have included mechanistic models, based on the infection patterns seen in the population and the deployment of protective measures, including social distancing and vaccination [8], and statistic models, based mostly on clinical reports and supporting data, including social mobility dynamics, weather reports, pollution levels, and even social media activity, among others [9]. Recent studies have taken advantage of machine learning approaches to integrate large datasets (sometimes encompassing more than one country) and develop advanced regression models, as the capacity of linear models to reflect on infection patterns has proven limited [10]. Neural network-based models, such as artificial neural networks (ANNs), bidirectional long short-term memory (LSTM), an adaptive neuro-fuzzy inference system (ANFIS), an autoregressive integrated moving average (ARIMA), and multilayer perceptron (MLP), have been trained on clinical reports published by public health authorities to predict future cases, reaching R2 coefficients of determination (R2) between 0.62 and 1 and a Mean Absolute Percentage Error (MAPE) generally below 10% [10].

While most studies have used reported clinical data, integrating parallel data to develop a fuller image of public health status in a population might be useful to develop better epidemiological models. In this regard, data arising from wastewater-based surveillance (WBS) might be a useful resource, as it allows for time- and resource-efficient study of a population by tracking and quantifying specific biomarkers in wastewater samples, which are representative of the entire population within the catchment area of the sampling point of interest [11]. Moreover, WBS data show potential for risk assessment models, as they can represent cases regardless of the level of individual clinical cases or the onset of symptoms [12]. Moreover, as studies have demonstrated that COVID-19 has an incubation period of around 5 to 7 days [13], increases in the load of viral genetic materials in wastewater samples may increase noticeably before the onset of epidemiological waves. However, it is important to note that WBS data should not be analyzed in isolation and should be integrated into epidemiological reports to obtain valuable information, as high biomarker variability, the lack of a standardized normalization technique, and interference due to high matrix complexity are ongoing challenges for the interpretation of WBS data for public health assessment [14].

Following this line, this work reports on the integration of WBS data obtained at key sampling points in the Monterrey Metropolitan Area (MMA, 5,341,171 inhabitants) and Mexico City (CDMX, 21,804,515 inhabitants) between January 2021 and June 2022 into simple statistical prediction models based on machine learning algorithms. Two main approaches were followed: the clustering of weekly data points as above or below a threshold indicative of a COVID-19 outbreak, and regression models offering an estimate of the seven-day average new reported daily COVID-19 cases adjusted for population size.

## 2. Materials and Methods

### 2.1. Data Acquisition

Data on SARS-CoV-2 viral load in wastewater samples (presented as viral genome copies per liter of wastewater) originating from the MMA and CDMX was compiled from the dataset previously obtained from the WBS platform operated by our laboratory between January 2021 and March 2022 [15]. Briefly, 1 L grab samples were obtained weekly from designated sampling sites encompassing both facilities of the largest private higher education institution in Mexico and wastewater treatment plants (WWTPs), transported to the central laboratory and concentrated using a polyethylene glycol- and NaCl-based method [16]. RNA was extracted using the DNA/RNA Magnetic Bead Kit (IDEXX, Westbrook, Maine) adapted for automation using a KingFisher™ Flex instrument (Thermo Fisher, Waltham, MA, USA), and the SARS-CoV-2 viral load was determined through the SARS-CoV-2 RT-qPCR Test kit for wastewater samples (IDEXX) on a QuantStudio 5 instrument (Applied Biosystems, Waltham, MA, USA). Sampling sites within the two university campuses (one located in the MMA and the other in CDMX) were grouped. The weekly data included (1) the maximum viral load detected across all sampling sites for each campus and (2) the percentage of buildings where viral load was detected (calculated as the ratio of samples testing positive for SARS-CoV-2 genetic material). For surveillance at WWTPs, data from one WWTP per city were included in the study. As such, only the total detected viral load was recorded. As observed in a previous study [17], the sampling rate on both campuses (expressed as the number of samples obtained from a given campus each week divided by the total number of sampling sites recorded at the campus) varied significantly across the study period and have an impact on the representativity of the data coming from the surveillance platform. To control this, data points where the percentage of sampled buildings on either campus fell below 20% were filtered out of the dataset. Furthermore, data points corresponding to weeks in which no sampling at the WWTP was reported were also excluded.

Daily new COVID-19 cases for the state of Nuevo León (where the MMA is located) and CDMX were obtained from the dashboard published by the National Council of Humanities, Sciences, and Technologies (CONAHCYT, Mexico), using data provided by the General Direction of Epidemiology, part of the Mexican Department of Health (available at https://datos.covid-19.conacyt.mx/, accessed on 9 February 2024). As wastewater sampling took place weekly and, given the observed inconsistencies in clinical reporting (for instance, cases across different days being added in a single day while the others were reported as zero), a seven-day average of daily new cases was calculated for each week. To ensure consistency in data analysis, daily new cases were normalized to cases per 100,000 inhabitants.

Urban mobility data for Nuevo León and CDMX were sourced from the COVID-19 Community Mobility Reports published by Google in 2021 and 2022 (available at https://www.google.com/covid19/mobility/, accessed on 9 February 2024). An average of the six reported mobility parameters was calculated to serve as an overall mobility indicator, and a seven-day average was applied weekly.

After data compilation, the data points included four predictors: (1) the maximum viral load detected across all sampling sites for each campus (Max_Campus), (2) the viral load in the WWTP (WBE_Dato), (3) the rate of positive buildings described previously (x_EdPositivos), and (4) the average of the six reported mobility parameters (AVG_Mobility). The seven-day average daily new COVID-19 cases per 100,000 inhabitants as a response. A data point corresponds to data from a single week. The complete dataset used in this study before filtering is provided in Table S1.

### 2.2. Definition of a Threshold to Identify Surges in Clinical Cases

To analyze the behavior of the obtained parameters throughout the study period and identify a suitable threshold for outbreak detection, principal component analysis (PCA) was conducted. First, wastewater surveillance and mobility data were normalized through unit variance scaling, taking the mean and standard deviation for each city. PCA was conducted via the singular value decomposition (SVD) method. To identify a potential threshold to effectively identify data points corresponding to increases in clinical reports, the scores obtained for principal components 1 and 2 were annotated based on whether the seven-day average of daily new COVID-19 reports from the week they corresponded to were above or below a specific threshold. The thresholds tested in the study varied between 1.5 and 5 daily new COVID-19 cases per 100,000 inhabitants. After plotting, ellipses were drawn to identify the area corresponding to a 95% confidence level for each of the two groups to visualize the behavior of the two groups for each threshold. An additional PCA plot was obtained by separating the filtered data points by city of origin (MMA or CDMX) to see if differences in the data collected at each location persisted after normalization.

Outbreak thresholds were further validated by conducting hierarchical clustering scaled predictors using Euclidean distances and average linkage. Two clusters were obtained, and the data points in each cluster were sorted into two groups based on whether the number of cases in the corresponding week was above or below a threshold between 1.5 and 5 daily new COVID-19 cases per 100,000 inhabitants. The larger cluster was labeled as the group to which the majority of the data points belonged: above the threshold (labeled as 1) or below the threshold (labeled as 0). The smaller cluster received the opposite label to the larger one. After labeling, the accuracy rate across both clusters was used to evaluate each threshold. Finally, the selected threshold was used to create a clustergram.

### 2.3. Classification-Based Predictive Models

After defining a suitable threshold, a column was appended to the database identifying if the seven-day average of new COVID-19 reports each week was above or below the threshold and divided randomly into a training set and a testing set. Clustering algorithms were developed using the “fitcauto” function in Matlab R2024a, running a Bayesian optimizer for 50 iterations for optimization with the default option to fold the training data five times for cross-validation. During training, the model assessed whether clinical cases were above or below the set threshold during the same week wastewater sampling was conducted. Only the training subset was fed into the learning function. Classification model performance was evaluated by calculating its accuracy rate, sensitivity, specificity, and Youden’s index on both training and testing subsets. The process was repeated 50 times, after which a single model (denominated F0) was chosen based on the performance metrics calculated.

**Figure 1:**
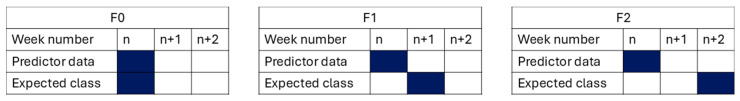
Displacement of the expected class for F0, F1, and F2.

After a satisfactory model was obtained, the forecasting ability of the wastewater surveillance platform was tested. For this, all predictor variables were matched to the response displaced one (F1) and two (F2) weeks into the future, as shown in Figure 1. Model performance in F1 and F2 was evaluated using the same metrics mentioned above.

### 2.4. Regression-Based Predictive Models

Two regression models to estimate COVID-19 incidence using the available predictors were explored: a simple linear regression model and machine learning-based regression.

For simple linear regression, an initial model was obtained using all four predictors using the “fitlm” function in Matlab R2024a, and the coefficient with the largest associated *p*-value was eliminated from the model. This process was repeated until a regression model where all coefficients were significantly different from zero (*p*-values below 0.05) was obtained. Model performance was assessed using Root Mean Square Error (RMSE), R^2^ coefficient, and Mean Absolute Percentage Error (MAPE).

For machine learning regression, data points were randomly divided into training and test subgroups. Models were constructed using the “fitrauto” function in Matlab R2024a, which optimizes training hyperparameters through Bayesian optimization, or up to 30 iterations with the default 5-fold cross-validation option, exploring all available learners. The process was repeated 50 times, after which a single model was chosen by comparing its performance to the simple linear regression model and consistency in the performance on both data subsets (training and test).

## 3. Results

### 3.1. PCA and Heatmap

**Figure 2:**
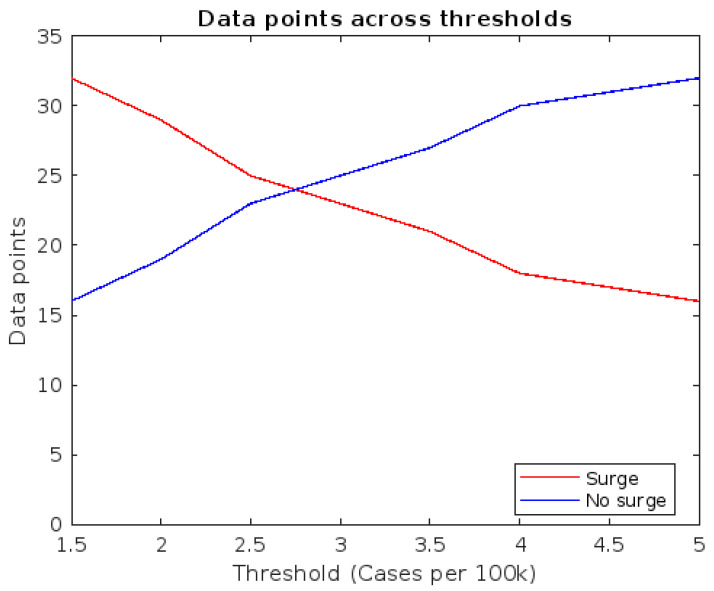
Classification of the 48 data points based on the explored threshold values. Data points above the threshold in red, and below the threshold in blue.

After filtration, the resulting database included 48 data points: 29 from the MMA and 19 from CDMX. The main limiting factor in the MMA was the large number of sampling sites encompassed within the studied university campus, which limited our ability to reliably sample them all across the entire study period. In CDMX, while the studied university campus was significantly smaller, the period in which sampling at WWTPs was conducted was significantly shorter than in the MMA. Figure 2 shows the number of data points above and below each of the thresholds investigated.

**Figure 3:**
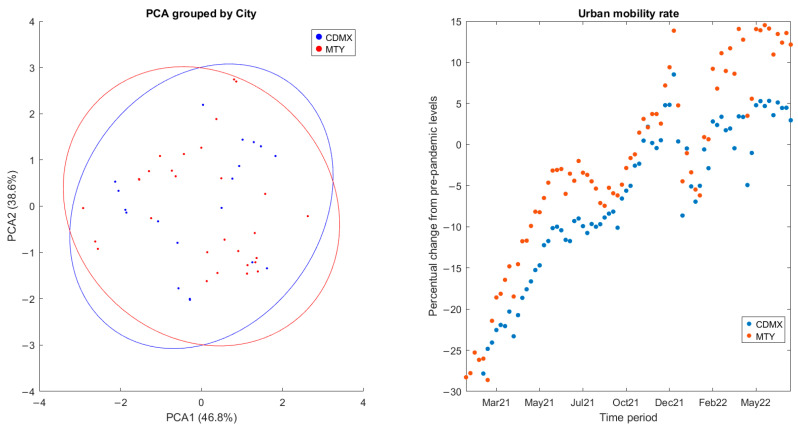
Principal component analysis for the database used in this study, classified by city (**left**). Changes in urban mobility during the study period, expressed as a percentage change from the pre-pandemic baseline (**right**).

PCA plots for the data classified by city of origin are presented in Figure 3. As expected, no clear separation between the two cities is observed when plotting PC1 (accounting for 46.8% of variance) against PC2 (accounting for 38.6% of variance), indicating that transmission dynamics are likely similar after controlling for population size. In fact, the average change in mobility presented in the COVID-19 Community Mobility Reports published by Google during the study period, reported as a percentage change from a pre-pandemic baseline, shows similar behaviors in both cities. This is to be expected since the interconnectedness of population centers due to economic globalization has been noted as a driver in the rapid spread of SARS-CoV-2 [18]. Overall, similar patterns of surges and reductions in clinical cases and comparable containment measures can be seen in both cities. As a result, data points can be reliably combined for modeling after controlling for the different population sizes. This is consistent with reports by [19], where surges in COVID-19 cases at regional and country levels across 2020 and 2021 were found to appear at similar times and have similar durations.

**Figure 4:**
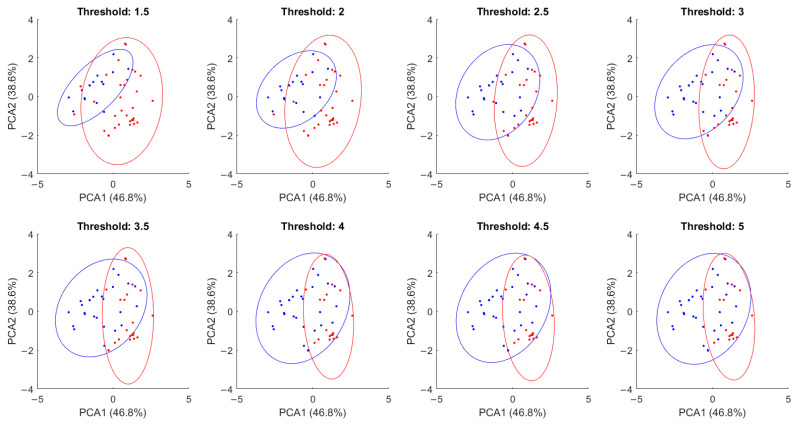
PCA plots for all data points grouped according to the investigated thresholds.

**Figure 5:**
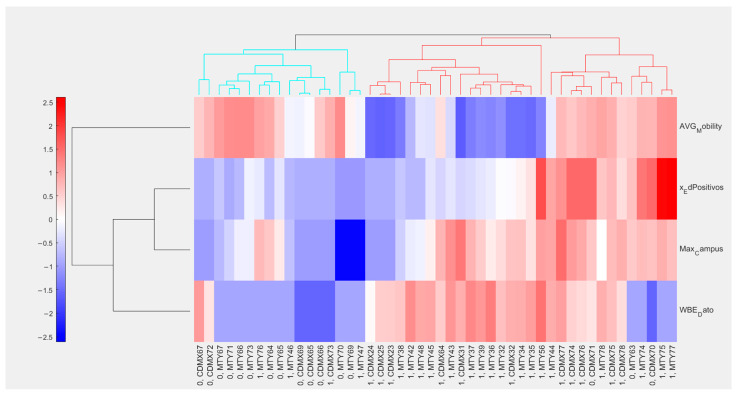
Clusterram presents correlations between the parameters integrated into the database. Clustering in the columns was conducted through Euclidean distances and average linkage. For columns, distances were calculated through correlation.

PCA plots for the filtered data points, grouped using thresholds between 2.5 and 6 cases per 100,000 inhabitants, are presented in Figure 4. In all cases, principal components 1 (explaining 46.8% of variance) and 2 (38.6% of variance) were used. The ellipses are drawn around the area of 95% confidence for each group overlapped in all cases, although the area of overlap decreased as the threshold went higher. This indicates that differential patterns in the parameters of interest exist, although some degree of confusion in the model is to be expected. This was further observed using hierarchical clustering, as presented in Table 1. The highest accuracy rate using the established distance and linkage configuration was 85.42% when using 1.5 daily new cases per 100,000 inhabitants. Accounting for population sizes, that is equivalent to 80 cases in Nuevo León and 327 in Mexico City. Figure 5 presents the obtained clustergram, with observations presented in the columns, while predictor variables are presented in the rows.

For the predictor variables, presented in the rows, distances were calculated using correlation coefficients to observe similar behaviors between them. The viral load found in samples from the WWTPs, the maximum viral load found on any sample from the university campus studied in each city, and the rate of sampling sites where viral load was detected on each campus. Meanwhile, the average urban mobility and the percentage of tested buildings on each campus showed the inverse behavior and were placed in a separate cluster.

Interestingly, some data points showed higher rates of positive buildings while sampling coverage remained close to the average on each campus, but no significant surge in clinical reports was observed. These data points come from May and June 2022, after urban mobility for both cities went back to pre-pandemic levels, and are likely based on the anticipation of a surge in cases that led to the fifth wave of COVID-19 cases in Mexico, which took place during the summer of 2022 [20]. This is consistent with our previous report, where we demonstrated that Omicron variants circulated in wastewater from university campuses across Mexico between January and March 2022 [21]. This observation supports the potential of decentralized, building-by-building WBS platforms in high-affluence areas, such as university campuses, as the rate of positive samples taken each week can provide relevant information for decision-making when overall population dynamics are accounted for. Similar observations were reported by Wolken et al. [22], from data using a similar WBS platform across pre-K-12 schools in Houston operated between December 2020 and May 2022.

### 3.2. Cluster-Based Risk Assessment Model

For the training and testing of cluster-based risk assessment models, 33/48 and 15/48 of the total observations in the dataset, combining data from both the MMA and CDMX, were used, respectively. Performance metrics for all 50 trials are presented in Table S2. The model obtained in trial number 7 was chosen given its high accuracy and consistent performance on both training and testing data. Model performance metrics are presented in Table 2. In short, the model had an accuracy rate of 87.88% for the training subset and 85.71% for the test subset, slightly higher than the 85.42% obtained using hierarchical clustering.

**Figure 6:**
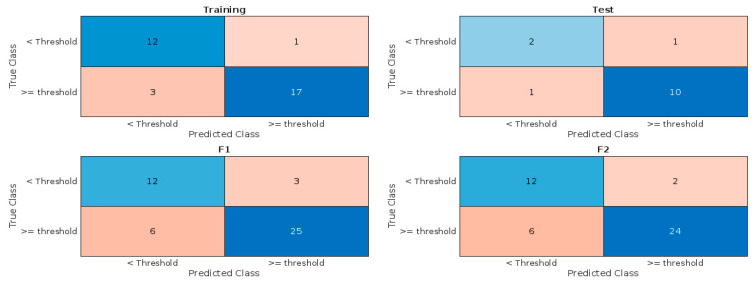
Confusion charts obtained by the clustering-based model, predicting surges in clinical reports and spaces in between surges, for the training partition of the current-week data, the testing partition, and forecasts one (F1) and two (F2) weeks into the future.

The confusion charts presented in Figure 6 indicate that the model generalizes well, using general trends in WBS data to separate data points linked to increased clinical reports and those linked to periods between surges instead of amplifying unrelated background variances. However, the observed Youden’s index decreased slightly, from 0.77 in the training subset to 0.58 in the test subset. After the selected model proved satisfactory, it was used to provide forecasts one and two weeks into the future using the same dataset to investigate whether the trends observed during clustering could be used for risk assessment. As seen in Table 2, predictions one week into the future showed an accuracy rate of 80.43% and a Youden of 0.61. Predictions two weeks into the future had a similarly strong accuracy rate (81.82%), and Youden index (0.66). However, the model had a noticeably higher tendency to yield false negative predictions (6/46 for one-week predictions and 6/44 for two-week predictions) when compared to predictions in F0 (4/48).

The difference is likely since the SARS-CoV-2 incubation period has been found to be between 5 and 7 days, shorter than initially expected [13], reducing the window of time in which increased viral loads could be found in wastewater samples before surges. Still, this supports the potential that WBS platforms can provide valuable information on epidemiological trends, which can be used for decision-making by public health authorities [23]. Moreover, sampling approaches such as this one, combining data from WWTPs and decentralized sampling points located at high-affluence sites (in this case, at college campuses), can be used for more focalized preventive and containment measures, allowing for continued operation in such sites while keeping them from becoming transmission hotspots [24]. In any case, it is important to remark that WBS data cannot be used in isolation nor are they a substitute for individualized clinical testing; rather, as discussed by Islam et al. [14], these approaches should be conducted collaboratively to obtain more robust datasets.

### 3.3. Regression-Based Predictive Models

The obtained linear regression model only included two predictors: the weekly rate of positive buildings (x_EdPositivos) and the average variation in urban mobility from the pre-pandemic baseline (AVG_Mobility). All other predictors were taken out of the model, as they were not significantly different from zero. All coefficients obtained in each regression model and their associated *p*-values are presented in Table S2. The final model showed a RMSE of 10.02 (expressed as daily new cases per 100,000 inhabitants), an R^2^ of 0.40, and a MAPE of 398.78%.

**Figure 7:**
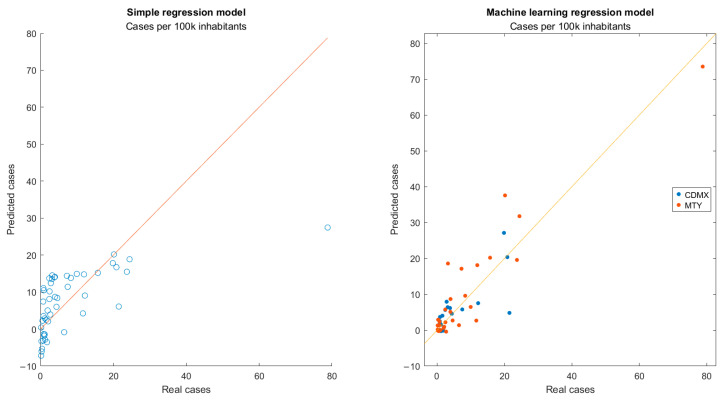
Number of new daily COVID-19 cases predicted by the regression-based model compared with the actual clinical reports. The line corresponds to the ideal linear relation one to one in predicted and real cases.

For the training and testing of machine learning regression models, 33/48 and 15/48 of the total observations were used in the dataset, respectively. Performance metrics for each of the 50 models obtained are presented in Table S4. Model 43 was chosen given the consistent R^2^ values obtained for both the training (0.89) and test (0.80) subsets, allowing for RMSE (4.64 for training, 6.86 for test) and MAPE (159.90% and 76.82%, respectively) noticeably lower than those obtained using traditional linear regression. Performance metrics for all regression models are presented in Table 3, while a plot of the predicted response against the actual weekly average of daily new COVID-19 cases for both models is presented in Figure 7.

Interestingly, in data points where the actual responses were below 5 cases per 100,000 inhabitants, the linear regression model tended to overestimate cases based on viral loads in wastewater samples, while it underestimated when the actual responses were above 5 cases per 100,000 inhabitants. A possible explanation for this indicates two main shortcomings in the dataset. The first is its size, as observations where campus coverage was below 20% and no corresponding sampling at the city’s selected WWTP was reported were filtered out of the database before training. As described in a previous report [17], reduced sampling rates limited the capacity of the surveillance platform to accurately identify potential surges in COVID-19 incidence. This is clearly illustrated here by the data point corresponding to data from the MMA during week 56 (January 2023) remained as an outlier for both models, likely as a result of an increase in transmission rates observed during the holidays. Moreover, insufficient sampling was conducted in the weeks prior to and after this point, due to the winter break at the institution and restricted laboratory activities in January and early February 2023 due to the fourth wave of contagions in Mexico [25], which made the integration of this particular data point into both models difficult. Second, while urban mobility could be accounted for, no direct data on campus occupancy or clinical testing within the institution were available to the team. Integrating such parameters into the model may increase the variance in the responses explained by the model. While some of these shortcomings could be addressed using non-linear regression learners, overfitting remains a concern in such models, especially when using datasets published by public health authorities, which tend to be incomplete [26].

The models reported here show significantly lower predictive performance when compared to those reviewed by Ghafouri-Fard et al. [10], which offer forecasts based on previous clinical reports, weather data, and even internet search history with R2 values between 0.65 and 1 and MAPE values below 10%. However, the use of data from WBS platforms for predictive models has proven significantly more difficult. In a study by Lai et al. [27], investigating the potential of time series machine learning to make forecasts of COVID-19 cases using both epidemiological reports WBS data from the states of Pennsylvania and Wyoming, the lowest MAPE values observed were still around 39%, with some WBS-based models still being outperformed by “naïve” models, which did not take WBS data into account for training. A similar approach by Ai et al. [28] recommends the use of long short-term memory to obtain highly generalizable models that could account for a higher degree of variance in the response (R2 of up to 0.81 in the test set), while also highlighting the importance of avoiding overfitting when using such learner algorithms. This is because the detection and quantification of viral loads in wastewater samples show a high level of variability due to unsteady wastewater flow in the sampling sites and degradation of genetic materials because of external factors, such as pH, temperature, enzymatic activity, and light exposure [29]. Taking these factors into account during feature engineering has proven relevant to the two studies mentioned previously, although data allowing for such fine-tuned predictive models were unavailable for the present study.

## 4. Discussion

This study evaluated simple machine learning models, specifically linear learners, for risk assessment using WBS data from university campuses and WWTPs in CDMX and MMA, the two largest cities in Mexico. These models demonstrated the ability to establish correlations between viral loads in wastewater samples and provide short-term forecasts that could support risk management and mitigation strategies in high-occupancy facilities, such as educational institutions or workplaces. However, their capacity for long-term forecasting is limited compared to more advanced models, such as long short-term memory regressions.

The dataset’s low dimensionality and limited size constrained the application of more sophisticated models. For instance, models incorporating environmental parameters that influence the degradation of genetic material—such as wastewater flow rates, pH, and temperature—have shown higher performance in other studies [27,28]. The absence of such parameters in our dataset highlights a critical limitation in capturing the full complexity of factors influencing viral load trends.

To improve model robustness and generalizability, future studies should integrate viral load data from a broader range of sampling sites across the country, alongside relevant epidemiological factors such as urban mobility, vaccination rates, and test positivity rates. This would enhance the models’ ability to represent trends on a national scale. Nevertheless, precautions against model overfitting remain essential, especially when employing non-linear approaches [26].

In any case, drawing correlations between the quantification of disease-related biomarkers and the prevalence of the disease of interest within a population remains an area of opportunity for developments in WBS. McMahan et al. [30] integrated wastewater measurements into a susceptible–exposed–infectious–recovered (SEIR) model, which led to an underreported estimation of the rate of COVID-19 cases, with roughly 11 unreported cases for each reported case, which closely matched previous estimates by public health officials for the area at the time of the study (15 unreported cases for each reported case). Melvin et al. [31] proposed a novel normalization and standardization process, the Melvin Index, to control the impact of site variability during sampling in qPCR-based SARS-CoV-2 genetic material quantification. Using this method, surges in clinical reports could be predicted over a period of up to 15–17 days using data from several sampling sites across the state of Minnesota, USA. Hewitt et al. [32] related the frequency of SARS-CoV-2 viral detection at a managed isolation and quarantine facility to the one seen at a WWTP during a window of time at which incidence was reportedly low. By relating both measures, they estimated the possibility of detecting SARS-CoV-2 genetic materials in wastewater from WWTPs that were representative of the overall population, which was 87% when prevalence in the population was at 0.01%.

Recently, Mohring et al. [33] reported on an approach for a finer estimate of COVID-19 cases from WBS data where a cohort was regularly followed during the study period using self-administered antigen tests. They reported the need to use both a scaling factor and a delay window to relate SARS-CoV-2 viral loads in wastewater samples with COVID-19 prevalence. Interestingly, the delay window they found, which was around 5 days, closely matches the incubation period of the virus [12].

The development of statistical models integrating WBS data for risk assessment will likely be useful during the first stages of future pathogen outbreaks as a first tool for decision-making if the modeled pathogen proves to have similar transmission routes to the novel pathogen of concern. For instance, knowledge obtained from modeling the COVID-19 pandemic could be useful in case of an outbreak of a highly transmissible, airborne, viral disease, such as a possible increase in zoonotic transmission of Influenza A H5N1, as it has been reported recently [34].

## 5. Conclusions

In this paper, a database resulting from the detection and quantification of SARS-CoV-2 viral loads in wastewater samples originating from university campuses and WWTPs in the MMA and CDMX were used to train both clustering and regression models built using linear learner algorithms to study trends in the evolution of daily new COVID-19 during the pandemic. While linear classification discriminant analysis could distinguish between wastewater data obtained during clinical report surges and periods between surges at 87.88% accuracy and the trends observed by the model could be used for forecasting, the performance of regression-based models remained limited due to low dimensionality in the data, as relevant environmental measurements for the determination of sample integrity, such as pH, temperature, or wastewater flow at the sampling sites, were not available. While the approach explored here can be used for simple risk assessment for the deployment of adequate prevention and containment strategies within high-affluence facilities, such as universities or workspaces, especially in the early stages of a possible epidemiological outbreak, further work toward integration of more robust datasets into more complex models, capable of long-term forecasting that could be used for future pathogens similar to COVID-19, is still needed.

## Figures and Tables

**Table 1:** Clustering accuracy in the investigated thresholds using Euclidean distances and average linkage.

Threshold			Accuracy
In Cases per 100,000 Inhabitants	Daily New Cases in Nuevo Leon	Daily New Cases in Mexico City	
1	53.22	218.05	77.08%
1.5	79.83	327.07	85.42%
2	106.44	436.09	83.33%
2.5	133.05	545.11	79.17%
3	159.66	654.14	79.17%
3.5	186.27	763.16	75.00%
4	212.88	872.18	68.75%

**Table 2:** Performance metrics for the obtained linear discriminant classification model for detection and forecasting of surges in COVID-19 cases using WBS data. F1 and F2 denote forecasts one and two weeks into the future, respectively.

	Accuracy	Sensitivity	Specificity	Youden
Training	0.8788	0.8500	0.9231	0.7731
Test	0.8571	0.9091	0.6667	0.5758
F1	0.8043	0.8065	0.8000	0.6065
F2	0.8182	0.8000	0.8571	0.6571

**Table 3:** Performance metrics of the regression models obtained to predict the weekly average of daily new COVID-19 cases using WBS data.

Model		RMSE	R^2^	MAPE
Simple linear regression		10.0227	0.4002	398.7772
Machine learning-based regression	Training	4.6434	0.8935	159.9020
	Test	6.8573	0.8042	76.8172

## Data Availability

Data supporting results are available in the Supplementary Materials. All code used for data analysis can be found at https://github.com/ArnArmenta/MARTEC_MachineLearning (accessed on 15 October 2024). For any other related information, please contact the corresponding author.

## Supplementary Materials

The following supporting information can be downloaded at: https://www.mdpi.com/article/10.3390/v17010109/s1, Table S1: complete database used in the study, Table S2: classification model performance, Table S3: linear regression coefficients, and Table S4: regression model performance.

## References

[1] Mingaleeva R.N., Nigmatulina N.A., Sharafetdinova L.M., Romozanova A.M., Gabdoulkhakova A.G., Filina Y.V., Shavaliyev R.F., Rizvanov A.A., Miftakhova R.R. (2022). Biology of the SARS-CoV-2 Coronavirus. Biochemistry.

[2] Bennett C.M., Vally H. (2024). The evolving epidemiology of SARS-CoV-2. Microbiol. Aust..

[3] Holmes E.C. (2024). The Emergence and Evolution of SARS-CoV-2. Annu. Rev. Virol..

[4] Manabe H., Manabe T., Honda Y., Kawade Y., Kambayashi D., Manabe Y., Kudo K. (2024). Simple mathematical model for predicting COVID-19 outbreaks in Japan based on epidemic waves with a cyclical trend. BMC Infect. Dis..

[5] San Martín-López J.V., Mesa N., Bernal-Bello D., Morales-Ortega A., Rivilla M., Guerrero M., Calderón R., Farfán A.I., Rivas L., Soria G. (2023). Seven Epidemic Waves of COVID-19 in a Hospital in Madrid: Analysis of Severity and Associated Factors. Viruses.

[6] Ritchie H., Mathieu E., Rodés-Guirao L., Appel C., Giattino C., Hasell J. Coronavirus Pandemic (COVID-19). Our World in Data, 2024. https://ourworldindata.org/coronavirus.

[7] Jewell N.P., Lewnard J.A., Jewell B.L. (2020). Predictive Mathematical Models of the COVID-19 Pandemic: Underlying Principles and Value of Projections. JAMA.

[8] Desikan R., Padmanabhan P., Kierzek A.M., Van Der Graaf P.H. (2022). Mechanistic Models of COVID-19: Insights into Disease Progression, Vaccines, and Therapeutics. Int. J. Antimicrob. Agents.

[9] Wolkewitz M., Lambert J., Von Cube M., Bugiera L., Grodd M., Hazard D., White N., Barnett A., Kaier K. (2020). Statistical Analysis of Clinical COVID-19 Data: A Concise Overview of Lessons Learned, Common Errors and How to Avoid Them. Clin. Epidemiol..

[10] Ghafouri-Fard S., Mohammad-Rahimi H., Motie P., Minabi M.A.S., Taheri M., Nateghinia S. (2021). Application of machine learning in the prediction of COVID-19 daily new cases: A scoping review. Heliyon.

[11] Jiménez-Rodríguez M.G., Silva-Lance F., Parra-Arroyo L., Medina-Salazar D.A., Martínez-Ruiz M., Melchor-Martínez E.M., Martínez-Prado M.A., Iqbal H.M.N., Parra-Saldívar R., Barceló D. (2022). Biosensors for the detection of disease outbreaks through wastewater-based epidemiology. TrAC Trends Anal. Chem..

[12] Scott L.C., Aubee A., Babahaji L., Vigil K., Tims S., Aw T.G. (2021). Targeted wastewater surveillance of SARS-CoV-2 on a university campus for COVID-19 outbreak detection and mitigation. Environ. Res..

[13] Zaki N., Mohamed E.A. (2021). The estimations of the COVID-19 incubation period: A scoping reviews of the literature. J. Infect. Public Health.

[14] Islam Md A., Hossen F., Rahman Md A., Sultana K.F., Hasan M.N., Haque Md A., Sosa-Hernández J.E., Oyervides-Muñoz M.A., Parra-Saldívar R., Ahmed T. (2023). An opinion on Wastewater-Based Epidemiological Monitoring (WBEM) with Clinical Diagnostic Test (CDT) for detecting high-prevalence areas of community COVID-19 infections. Curr. Opin. Environ. Sci. Health.

[15] Sosa-Hernández J.E., Oyervides-Muñoz M.A., Melchor-Martínez E.M., Driver E.M., Bowes D.A., Kraberger S., Lucero-Saucedo S.L., Fontenele R.S., Parra-Arroyo L., Holland L.A. (2022). Extensive Wastewater-Based Epidemiology as a Resourceful Tool for SARS-CoV-2 Surveillance in a Low-to-Middle-Income Country through a Successful Collaborative Quest: WBE, Mobility, and Clinical Tests. Water.

[16] Sapula S.A., Whittall J.J., Pandopulos A.J., Gerber C., Venter H. (2021). An optimized and robust PEG precipitation method for detection of SARS-CoV-2 in wastewater. Sci. Total Environ..

[17] Armenta-Castro A., Oyervides-Muñoz M.A., Aguayo-Acosta A., Lucero-Saucedo S.L., Robles-Zamora A., Rodriguez-Aguillón K.O., Ovalle-Carcaño A., Parra-Saldívar R., Sosa-Hernández J.E. (2024). Academic institution extensive, building-by-building wastewater-based surveillance platform for SARS-CoV-2 monitoring, clinical data correlation, and potential national proxy. medRxiv.

[18] Jeanne L., Bourdin S., Nadou F., Noiret G. (2022). Economic globalization and the COVID-19 pandemic: Global spread and inequalities. GeoJournal.

[19] Bali Swain R., Lin X., Wallentin F.Y. (2024). COVID-19 pandemic waves: Identification and interpretation of global data. Heliyon.

[20] Taboada B.I., Zárate S., García-López R., Muñoz-Medina J.E., Gómez-Gil B., Herrera-Estrella A., Sanchez-Flores A., Salas-Lais A.G., Roche B., Martínez-Morales G. (2023). SARS-CoV-2 Omicron variants BA.4 and BA.5 dominated the fifth COVID-19 epidemiological wave in Mexico. Microb. Genom..

[21] Aguayo-Acosta A., Oyervides-Muñoz M.A., Rodriguez-Aguillón K.O., Ovalle-Carcaño A., Romero-Castillo K.D., Robles-Zamora A., Johnson M., Parra-Saldívar R., Sosa-Hernández J.E. (2024). Omicron and Delta variant prevalence detection and identification during the fourth COVID-19 wave in Mexico using wastewater-based epidemiology. IJID Reg..

[22] Wolken M., Sun T., McCall C., Schneider R., Caton K., Hundley C., Hopkins L., Ensor K., Domakonda K., Kalvapalle P. (2023). Wastewater surveillance of SARS-CoV-2 and influenza in preK-12 schools shows school, community, and citywide infections. Water Res..

[23] Polo D., Quintela-Baluja M., Corbishley A., Jones D.L., Singer A.C., Graham D.W., Romalde J.L. (2020). Making waves: Wastewater-based epidemiology for COVID-19—Approaches and challenges for surveillance and prediction. Water Res..

[24] Gonçalves J., Torres-Franco A., Rodriguéz E., Diaz I., Koritnik T., Silva P.G.D., Mesquita J.R., Trkov M., Paragi M., Muñoz R. (2022). Centralized and decentralized wastewater-based epidemiology to infer COVID-19 transmission—A brief review. One Health.

[25] De La Cruz-Hernández S.I., Álvarez-Contreras A.K. (2022). Omicron Variant in Mexico: The Fourth COVID-19 Wave. Disaster Med. Public Health Prep..

[26] Peng Y., Nagata M.H. (2020). An empirical overview of nonlinearity and overfitting in machine learning using COVID-19 data. Chaos Solitons Fractals.

[27] Lai M., Cao Y., Wulff S.S., Robinson T.J., McGuire A., Bisha B. (2023). A time series based machine learning strategy for wastewater-based forecasting and nowcasting of COVID-19 dynamics. Sci. Total Environ..

[28] Ai Y., He F., Lancaster E., Lee J. (2022). Application of machine learning for multi-community COVID-19 outbreak predictions with wastewater surveillance. PLoS ONE.

[29] Parra-Arroyo L., Martínez-Ruiz M., Lucero S., Oyervides-Muñoz M.A., Wilkinson M., Melchor-Martínez E.M., Araújo R.G., Coronado-Apodaca K.G., Velasco Bedran H., Buitrón G. (2023). Degradation of viral RNA in wastewater complex matrix models and other standards for wastewater-based epidemiology: A review. TrAC Trends Anal. Chem..

[30] McMahan C.S., Self S., Rennert L., Kalbaugh C., Kriebel D., Graves D., Colby C., Deaver J.A., Popat S.C., Karanfil T. (2021). COVID-19 wastewater epidemiology: A model to estimate infected populations. Lancet Planet. Health.

[31] Melvin R.G., Hendrickson E.N., Chaudhry N., Georgewill O., Freese R., Schacker T.W., Simmons G.E. (2021). A novel wastewater-based epidemiology indexing method predicts SARS-CoV-2 disease prevalence across treatment facilities in metropolitan and regional populations. Sci. Rep..

[32] Hewitt J., Trowsdale S., Armstrong B.A., Chapman J.R., Carter K.M., Croucher D.M., Trent C.R., Sim R.E., Gilpin B.J. (2022). Sensitivity of wastewater-based epidemiology for detection of SARS-CoV-2 RNA in a low prevalence setting. Water Res..

[33] Mohring J., Leithäuser N., Wlazło J., Schulte M., Pilz M., Münch J., Küfer K.-H. (2024). Estimating the COVID-19 prevalence from wastewater. Sci. Rep..

[34] Dye C., Barclay W.S. (2024). Should we worry about a growing threat from “bird flu”?. BMJ.

